# Monolithic calcium phosphate/poly(lactic acid) composite versus calcium phosphate-coated poly(lactic acid) for support of osteogenic differentiation of human mesenchymal stromal cells

**DOI:** 10.1007/s10856-016-5666-9

**Published:** 2016-01-19

**Authors:** Zeinab Tahmasebi Birgani, Clemens A. van Blitterswijk, Pamela Habibovic

**Affiliations:** Department of Tissue Regeneration, MIRA Institute for Biomedical Technology and Technical Medicine, University of Twente, P.O. Box 217, 7500 AE Enschede, The Netherlands; MERLN Institute for Technology-Inspired Regenerative Medicine, Maastricht University, P.O. Box 616, 6200 MD Maastricht, The Netherlands

## Abstract

Calcium phosphates (CaPs), extensively used synthetic bone graft substitutes, are often combined with other materials with the aim to overcome issues related to poor mechanical properties of most CaP ceramics. Thin ceramic coatings on metallic implants and polymer-ceramic composites are examples of such hybrid materials. Both the properties of the CaP used and the method of incorporation into a hybrid structure are determinant for the bioactivity of the final construct. In the present study, a monolithic composite comprising nano-sized CaP and poly(lactic acid) (PLA) and a CaP-coated PLA were comparatively investigated for their ability to support proliferation and osteogenic differentiation of bone marrow-derived human mesenchymal stromal cells (hMSCs). Both, the PLA/CaP composite, produced using physical mixing and extrusion and CaP-coated PLA, resulting from a biomimetic coating process at near-physiological conditions, supported proliferation of hMSCs with highest rates at PLA/CaP composite. Enzymatic alkaline phosphatase activity as well as the mRNA expression of bone morphogenetic protein-2, osteopontin and osteocalcin were higher on the composite and coated polymer as compared to the PLA control, while no significant differences were observed between the two methods of combining CaP and PLA. The results of this study confirmed the importance of CaP in osteogenic differentiation while the exact properties and the method of incorporation into the hybrid material played a less prominent role.

## Introduction

To overcome issues related to the use of natural bone grafts [1, 2] and to satisfy a rapidly increasing need for successful and affordable strategies to treat damaged and diseased bone tissue [2, 3], significant efforts are currently invested in developing synthetic alternatives to natural bone. While all three main material types, i.e. metals, ceramics and polymers, as well as their combinations have been used as bone graft substitutes, calcium phosphate (CaP) ceramics, varying in chemistry (hydroxyapatite, tricalcium phosphate, brushite, octacalcium phosphate, etc.) [4] and mode of application (sintered bulk ceramics, particles, injectable cements, etc.) [5, 6] are the most widely used materials, owing to their chemical resemblance to bone mineral [7]. CaPs possess excellent biocompatibility in osseous environment [1, 2, 5, 8], and more importantly, they are generally accepted as osteoconductive materials [9, 10], with a subpopulation even being osteoinductive [11–13]. CaPs, however, suffer from intrinsic brittleness, which is an important limiting factor, particularly in load-bearing applications [14, 15]. To overcome this issue, CaP ceramics have been combined with other materials, in particular polymers, in the bulk [14–25] or as surface coatings [8, 26]. For example, CaPs have been used to develop monolithic composites with poly(α-esters) such as poly(lactic acid) (PLA), poly(glycolic acid) (PGA) and their copolymers (PLGA) [17–20], protein based polymers including collagen [21] and gelatin [22, 23], polysaccharides like chitosan [24] as well as synthetic co-polymers such as poly(ethylene oxide terephthalate)/poly(butylene terephtalate) (PEOT-PBT) [25]. Alternatively to conventional composites, physical assembly of the individual components [25] has been used to develop polymer-ceramic hybrids. Concerning coating techniques, classical methods for coating CaPs on substrates, such as plasma-spraying, have mainly been used to coat non-degradable permanent metallic implants to improve their bioactivity [8, 26–29], for example in total hip arthroplasty. Nevertheless, examples of more subtle coating techniques exist, which are suitable for coating thermally less stable materials including polymers, such as biomimetic coating process [30–32], radio frequency (RF) magnetron sputtering [33], or pulse laser deposition [34].

Properties of a hybrid material, as well as its biological performance, are dependent on the properties of each of the components, as well as on the way they are combined. For example, degradation of a CaP/polymer composite depends on the physico-chemical properties of the ceramic (CaP phase, crystallinity, surface area, etc.), physico-chemical properties of the polymer (composition, molecular weight, level of crosslinking etc.) as well as the way they are integrated into the final product (solvent-based mixing, physical mixing, coating, etc.).

In the current study, we hypothesized that direct contact between the CaP component of a CaP/polymer hybrid material and the biological environment is beneficial for the bioactivity of the hybrid. To test this, we have produced PLA particles and coated them with a thin layer of CaP by immersion into a saturated CaP solution, and compared them to composite particles produced by the extrusion of a PLA/nano-sized CaP mixture. Upon characterization of both particle types, bone marrow-derived human mesenchymal stromal cells (hMSCs) were cultured on the two materials, followed by the assessment of their proliferation and differentiation towards the osteogenic lineage.

## Materials and methods

### Materials production

For this study, two hybrid materials consisting of CaP and PLA were produced: a monolithic PLA/CaP composite and a CaP-coated PLA. PLA without CaP served as control. The composite consisted of 50 wt% amorphous poly(d,l-lactic acid) (PLA) (Purasorb PDL05, Purac, MW: 59000 g mol^−1^) and 50 wt% nano-sized CaP apatite powder, prepared in-house using a wet precipitation method as was described previously [18–20]. Briefly, the HA was precipitated in a mixture of aqueous solutions of (NH_4_)_2_HPO_4_ and Ca(NO_3_)_2_·4H_2_O at a pH above 10. The resulting precipitate was allowed to age overnight, washed and finally resuspended in acetone and allowed to dry. The composite was produced by extrusion using a twin-screw extruder with conical non-converging screws (Artecs BV, Enschede, The Netherlands). The PLA and CaP powder were mixed for 5 min in the extruder at 150 °C using the screw rotation speed of 100 rpm. The composite was extruded in the form of rods, which were ground and sieved to obtain composite particles in the range of 0.5–1 mm.

PLA particles were obtained using the same procedure of extrusion, grinding and sieving as described above for the PLA/CaP composite. The particles were either left uncoated or were coated with a CaP layer via a two-step biomimetic method similar to the one described earlier [35, 36]. In short, the particles were first immersed in a concentrated Simulated Body Fluid (SBF 2.5x) with ionic content of 733.5 mM Na^2+^, 7.5 mM Mg^2+^, 12.5 mM Ca^2+^, 720 mM Cl^−^, 5 mM HPO_4_^2−^ and 21 mM HCO_3_^−^, under stirring at 37 °C for 3 days, with daily refreshment. In the second step, the particles were incubated with a calcium phosphate solution (CPS) consisting of 140 mM Na^2+^, 4 mM Ca^2+^, 2 mM HPO_4_^2−^ and 144 mM Cl^−^ (buffered at pH 7.4), while stirring at 37 °C for 3 days with two refreshments. The coated PLA particles were then washed three times with MilliQ water and dried at least overnight in an air oven at 37 °C.

### Material characterization

The surface morphology and elemental analysis of calcium, phosphorous and carbon were investigated on gold-sputtered PLA, PLA/CaP composite and CaP-coated PLA particles using scanning electron microscopy (SEM, XL-30 ESEM-FEG, Philips) in the secondary electron mode, coupled with energy dispersive X-ray spectroscopy analyzer (EDS, EDAX, AMETEK Materials Analysis Division) at the accelerator voltage of 10 keV and working distance of 10 mm. The chemical composition of various particles was characterized using Fourier transform infrared spectroscopy (FTIR, Perkin-Elmer Spectrum 1000) in transmission mode and X-ray diffraction method (XRD, PANaltytical X’Pert). Thermogravimetric analysis (TGA, STA 449 F3, NETZSCH) was performed in the temperature range of 35–1000 °C and the weight loss was calculated to determine the mineral content.

The release of Ca^2+^ and inorganic phosphate (Pi) ions from different particles was analyzed in simulated physiological solution (SPS) containing 137 mmol L^−1^ Na+, 177 mmol L^−1^ Cl^−^, 50 mmol L^−1^ HEPES in MilliQ water and buffered at pH 7.3 over a period of 3 months. 100 ± 1 mg of PLA, PLA/CaP composite or CaP-coated PLA particles were precisely weighed and immersed in 5 ml SPS in plastic tubes. The tubes were then placed in a shaking water bath at 37 °C. At dedicated time points between 1 and 12 weeks, triplicates of each sample were removed from water bath and Ca^2+^ and Pi ion content of SPS was quantified using QuantiChrom™ Calcium assay kit (DICA-500, BioAssay Systems, USA) and QuantiChrom™ Phosphate assay kit (DIPI-500 BioAssay Systems, USA), respectively, according to manufacturer’s protocols.

### In vitro cell culture

hMSCs were isolated from bone marrow aspirates (5–20 ml) after written informed consent, as described previously [37, 38]. Briefly, aspirates were resuspended using 20-gauge needles, plated at a density of 5 × 10^5^ cells per cm^2^, and cultured in proliferation medium (consisting of α-MEM (Gibco) supplemented with 10 v/v % fetal bovine serum (Lonza), 2 mM l-glutamine (Gibco), 0.2 mM ascorbic acid (Sigma), 62.69 µg ml^−1^ penicillin, 100 µg ml^−1^ streptomycin (Gibco) and 1 ng ml^−1^ rhbFGF (AbDSerotec)). The medium was refreshed every 2–3 days. Cells were harvested at approximately 80 % confluency for subculture until passage 3.

In an earlier study, hMSCs that were isolated and expanded using this method were shown to be positive for CD29, CD44, CD105, and CD166 which are typical markers of hMSCs, and to possess the potential to differentiate into the osteogenic lineage in vitro and to induce new bone formation in vivo [38].

Approximately 100 µl of particles were placed in the wells of non plasma-treated 25-well plates with square-shaped bottom and sterilized with isopropanol prior to cell culture. To sterilize, the samples were washed three times with 100 % isopropanol followed by 15 min of drying inside the flow cabinet after each washing step. In the last step of sterilization, 100 % isopropanol was added to the samples and allowed to evaporate in the flow cabinet for at least 2 h. The samples were then washed twice with sterile PBS, followed by an overnight incubation in 1 ml basic medium (proliferation medium without rhbFGF) in a humidified atmosphere at 37 °C and 5 % CO_2_.

The particles were then collected in one corner of the wells and 200,000 hMSCs of passage 3 were seeded on the particles in approximately 50 µl of basic cell culture medium. This seeding method and density were optimized based on a preliminary study (data not shown). To maximize the attachment of the cells on the particles, the wells were tilted and cells were allowed to attach for 4 h. 1 ml of either basic medium or osteogenic medium (basic medium supplemented with 10 nM dexamethasone (Sigma)) was added to each well. The medium was refreshed every 3–4 days. Old medium was collected at each refreshment and the concentration of Ca^2+^ was determined using QuantiChrom™ calcium assay kit.

Total DNA amount was assessed with CyQuant Cell Proliferation Assay kit (Invitrogen) at day 7 and 14 after washing the samples with PBS. After 3 freeze/thaw cycles at −80° C, 500 µl lysis buffer (lysis buffer provided in the kit diluted in a buffer of NaCl-EDTA solution) was added to each well. The samples were ultra-sonicated and incubated at RT for 1 h. After centrifugation, 100 µl of the supernatant was mixed with the same volume of CyQuant GR dye in a 96 well micro-plate and incubated for 15 min. Fluorescence measurements for DNA quantification were performed at excitation and emission wavelengths of 480 and 520 nm, respectively, using a spectrophotometer (Perkin Elmer). ALP activity in the cultures was measured using a CDP-star kit (Roche Applied Science). 10 µl of the supernatant was mixed with 40 µl CDP-star reagent in a 96 well micro-plate and incubated for 30 min. After incubation, chemiluminescence measurements were completed at 466 nm. Results of the DNA assays are presented based on average µg of DNA detected in each condition. Results of ALP activity were normalized for DNA content of each culture and presented as the average of normalized ALP activity per µg of DNA for each condition.

Total RNA was isolated by using a combination of NucleoSpin^®^ RNA II isolation kit and Trizol method, in accordance with the manufacturer’s protocol. RNA was collected in RNAse-free water and the total concentration was measured using nano-drop equipment (ND1000 spectrophotomer, Thermo Scientific). The cDNA of the cultures were then prepared using iScript kit (Bio-Rad) according to the manufacturer’s protocol and diluted 10 times in RNAse-free water to be used for quantitative real-time PCR (qPCR). The qPCR measurements were completed using Bio-Rad equipment using Syber green I master mix (Invitrogen) and the primer sequences (Sigma), which are listed in Table 1. Expression of the osteogenic marker genes was normalized to GAPDH levels and fold induction was calculated by using the ΔΔC_T_ method. mRNA level of the desired genes in hMSCs cultured on treated tissue culture plates (TCPs) at the density of 10,000 cells cm^−2^ in basic cell culture medium for 7 days was also quantified and used for normalizing the results.

**Table 1:** Primer sequences of the osteogenic genes, the expression of which was investigated using qPCR analysis

Gene	Primer sequences
GAPDH (housekeeping gene)	5′-CCATGGTGTCTGAGCGATGT
	5′-CGCTCTCTGCTCCTCCTGTT
Alkaline phosphatase (ALP)	5′-TTCAGCTCGTACTGCATGTC
	5′-ACAAGCACTCCCACTTCATC
Runt-related transcription factor 2 (RUNX2)	5′-GGAGTGGACGAGGCAAGAGTTT
	5′-AGCTTCTGTCTGTGCCTTCTGG
Bone sialoprotein (BSP)	5′-TCCCGTTCTCACTTTCATA
	5′-CCCCACCTTTTGGGAAAAC
Bone morphogenetic protein 2 (BMP2)	5′-GCATCTGTTCTCGGAAAACCT
	5′-ACTACCAGAAACGAGTGGGAA
Osteopontin (OP)	5′-CCAAGTAAGTCCAACGAAAG
	5′-GGTGATGTCCTCGTCTGTA
Osteocalcin (OC)	5′-CGCCTGGGTCTCTTCACTAC
	5′-TGAGAGCCCTCACACTCCTC

### Statistical analysis

Statistical comparisons were performed using One-way Analysis of Variance (ANOVA) followed by a Tukey’s multiple comparison post hoc test. Error bars indicate one standard deviation. The level of significance was set at *P* < 0.05.

## Results

### Characterization of the hybrid materials

**Fig. 1:**
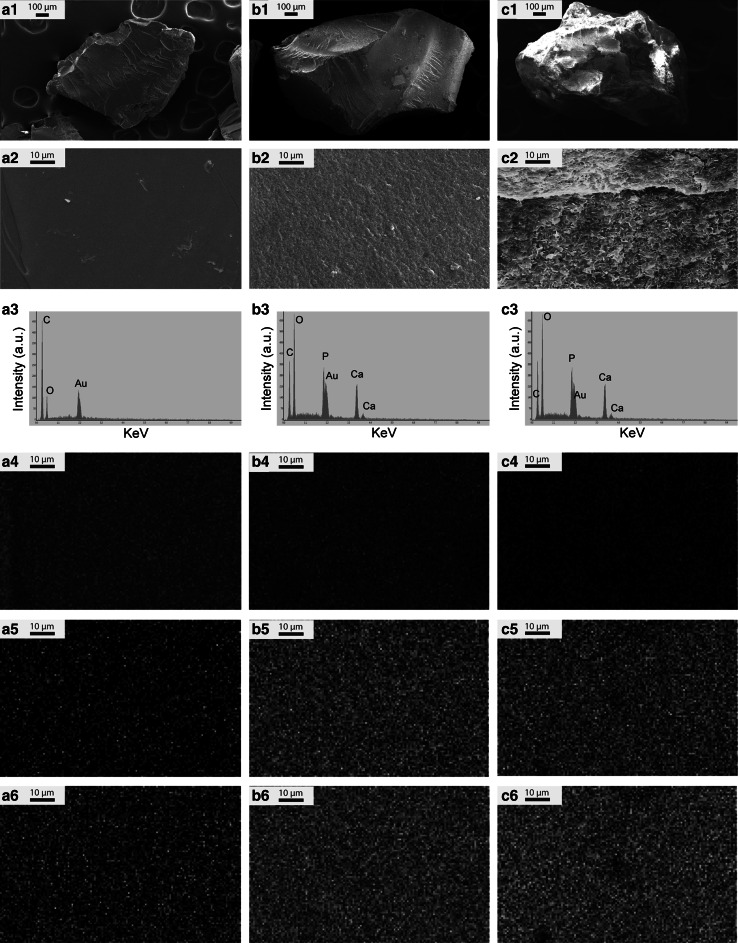
SEM images at low (**a1**, **b1**, **c1**) and high (**a2**, **b2**, **c2**) magnification, EDS spectra (**a3**, **b3**, **c3**), carbon- (**a4**, **b4**, **c4**), calcium- (**a5**, **b5**, **c5**) and phosphorus (**a6**, **b6**, **c6**) elemental map of PLA, PLA/CaP composite and CaP-coated PLA, respectively. The PLA particles exhibited smooth surface, while the surface of the PLA/CaP composite appeared rougher with a homogenous distribution of CaP powder within the PLA matrix. A uniform CaP layer consisting of small, plate-shaped crystals was observed on PLA-coated particles. It should be noted that gold (Au) peak was observed in all the EDS spectra because the samples were gold-sputtered before SEM/EDS analysis

Particles of both pure PLA and of the two hybrid materials were dense with an irregular shape and with a size ranging between 0.5 and 1 mm (Fig. 1a1–c1). While the surface of pure PLA appeared smooth (Fig. 1b1), PLA/CaP composite particles exhibited a more rough surface structure (Fig. 1b2). CaP-coated PLA particles were homogenously covered with a coating consisting of small plate-shaped crystals, giving it a rough surface morphology (Fig. 1b3).

The EDS spectrum of PLA particles exhibited only carbon (C) and oxygen (O) peaks (Fig. 1a3). A reduction in the intensity of C peak was observed in the EDS spectra of both PLA/CaP composite and CaP-coated PLA particles whereas the intensity of the O peak increased in these samples. Moreover, the EDS spectra of the two hybrid materials exhibited calcium and phosphorus peaks, demonstrating the presence of a CaP phase in these samples (Fig. 1b3–c3). The EDS elemental maps confirmed these results (Fig. 1a4–c6). A uniform distribution of calcium and phosphorus were observed in the elemental maps of composite and coated PLA particles showing a homogenous distribution of CaP in the polymer matrix and a uniform surface coating, respectively.

**Fig. 2:**
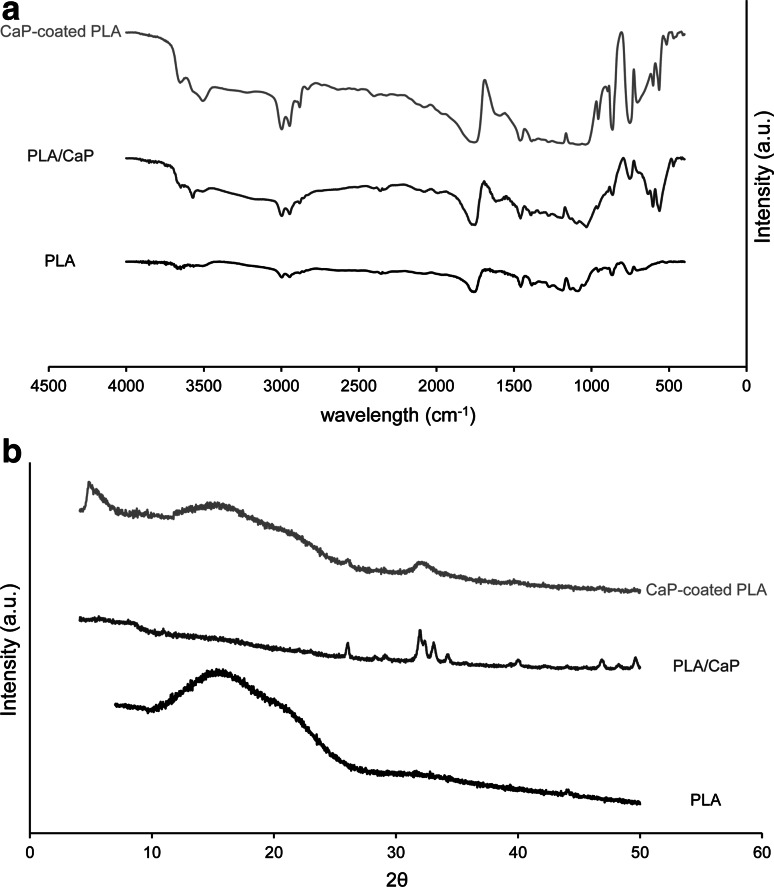
FTIR spectra (**a**) and XRD patterns (**b**) of the PLA, PLA/CaP and CaP-coated PLA particles. The FTIR spectra of the two hybrid materials exhibited additional bands demonstrating the presence of CaP. The XRD patterns suggested the presence of a crystalline HA in the composite and a mixture of OCP and apatite in the coated PLA

The FTIR spectra and the XRD patterns of the PLA, PLA/CaP composite and CaP-coated PLA are shown in Fig. 2. The spectrum of PLA showed peaks at approximately 1000–1100 cm^−1^, corresponding to stretching mode of C-O bond. The peaks of C–H bond in bending and stretching modes were observed at approximately 1370–1450 and 1950–2000 cm^−1^, respectively. A single peak that appeared at 1750 cm^−1^ is attributed to stretching mode of C=O bond [39, 40]. The PLA spectrum was in agreement with previously published data [18].

All peaks, typical of the PLA chemistry, were also found in the FTIR spectra of the two hybrid materials. A new band was observed around 1000 cm^−1^, probably due to the presence of PO_4_^3−^, the band of which appears at the similar wavelength [18, 19]. The bands at 560 and 604 cm^−1^ in the FTIR spectra of CaP-containing particles are also attributed to the P–O bond. Moreover, the hydroxyl bands at 635 and 3565 cm^−1^ were detected in the spectrum of PLA/CaP composite suggesting the apatitic phase [18, 19]. In the spectrum of the CaP-coated PLA, the hydroxyl band at 3565 cm^−1^ appeared as a small shoulder on the H_2_O band.

The XRD pattern of PLA particles did not show any distinguishable peaks, confirming the amorphous nature of the polymer. The XRD pattern of the PLA/CaP composite was in accordance with those obtained in previous studies, showing the presence of pure crystalline hydroxyapatite (HA) phase [18, 19]. The most intense diffraction lines at 2theta = 26, 31.9, 32.2, 33, 34.2, 29.9, 46.9 and 49.5°, are attributed (002), (211), (112), (300), (202), (130), (222), and (213) crystalline planes in HA [41]. The XRD pattern of CaP-coated PLA showed less intense and broader peaks compared to the one of the composite material, confirming the formation of a less crystalline CaP phase. The most intense peak was observed at approximately 4.9°, which is attributed (010) crystalline plane of octacalcium phosphate (OCP). The small peak found at 25.9° corresponds to (002) plane in both OCP and HA structure [41, 42]. The broad peak at about 32° is normally seen in the XRD pattern of OCP with a rather low intensity. The relatively high intensity of the peak at about 32° suggests the presence of an apatitic phase.

**Fig. 3:**
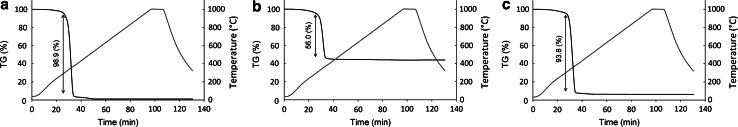
The TGA graphs showing weight loss of (**a**) PLA, (**b**) PLA/CaP and (**c**) CaP-coated PLA in the temperature range 35–1000 °C. A weight loss of 98.9, 56 and 93.8 % were measured for PLA, PLA/CaP and CaP-coated PLA, respectively

The TGA analysis (Fig. 3), performed in the temperature range 35–1000 °C, showed a single drop in weight for all materials. The weight loss occurred in the approximate temperature range of 300–400 °C and was calculated to be 98.9, 56 and 93.8 % for PLA, PLA/CaP composite and CaP-coated PLA particles, respectively.

**Fig. 4:**
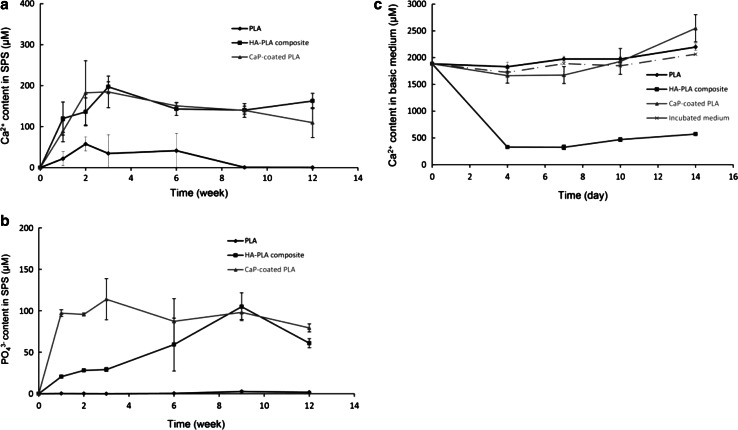
The Ca^2+^ (**a**) and Pi ion (**b**) concentrations in SPS and the Ca^2+^ (**c**) concentration in cell culture medium upon immersion of PLA, PLA/CaP and CaP-coated PLA particles. Both hybrid materials enriched the SPS solution with Ca^2+^ and Pi ions at earlier time points, followed by a slight decrease of concentrations of both ions at later time points. Immersion of CaP-coated PLA in cell culture medium resulted in an initial decrease of Ca^2+^ concentration, which was recovered at later time points. PLA/CaP composite particles depleted cell culture medium of the Ca^2+^ ions, at all time points analyzed

Cumulative release of Ca^2+^ and Pi ions over a period of 12 weeks was investigated upon immersion of polymeric and hybrid particles in CPS (Fig. 4a, b). A slight release of Ca^2+^ was measured in the solutions containing PLA particles at 1, 2, 3, and 6 weeks, possibly accidentally introduced to the solution during preparation, while no Pi ions were detected at any of the time points. A constant Ca^2+^ release to a maximum level of 200 µM was detected for PLA/CaP composite particles until week 3, after which a decrease to a concentration approximately 150 µM was observed. A linear increase in Pi ion concentration of PLA/CaP containing CPS was observed up to week 9, reaching a maximum concentration of 100 µM. At week 12, the Pi ion level substantially decreased to 60 µM. For the CaP-coated particles, an increasing release of Ca^2+^ up to 180 µM was observed until week 2. This concentration remained constant for another week, and then linearly decreased to approximately 100 µM at week 12. The Pi ion release profile of CaP-coated polymer showed an increase in concentration to 110 µM in week 3, followed by a drop to approximately 80 µM in the remaining experimental period.

The Ca^2+^ concentration in time was also examined in the cell culture medium over a 2-week period to investigate to which ionic concentrations the cells are exposed during culture (Fig. 4c). The Ca^2+^ concentration of the cell culture medium not containing any materials varied in the range of 1.7–2.0 mM over the 2-week period. Similar to the medium without materials, the Ca^2+^ concentration of the PLA-incubated medium showed variations in the range of 1.8–2.1 mM. A substantial decrease to approximately 300 µM was detected in Ca^2+^ concentration after immersion of PLA/CaP composite particles in cell culture medium for 4 days, followed by a gradual increase to 600 µM, at day 14. A slight decrease of approximately 200 µM was observed in the Ca^2+^ concentration of the medium containing CaP-coated PLA particles at the first time point, a concentration that was comparable to that of the medium not containing any material. The Ca^2+^ level then increased in time, up to 2.5 mM at day 14.

### Response of hMSCs to hybrid materials

**Fig. 5:**
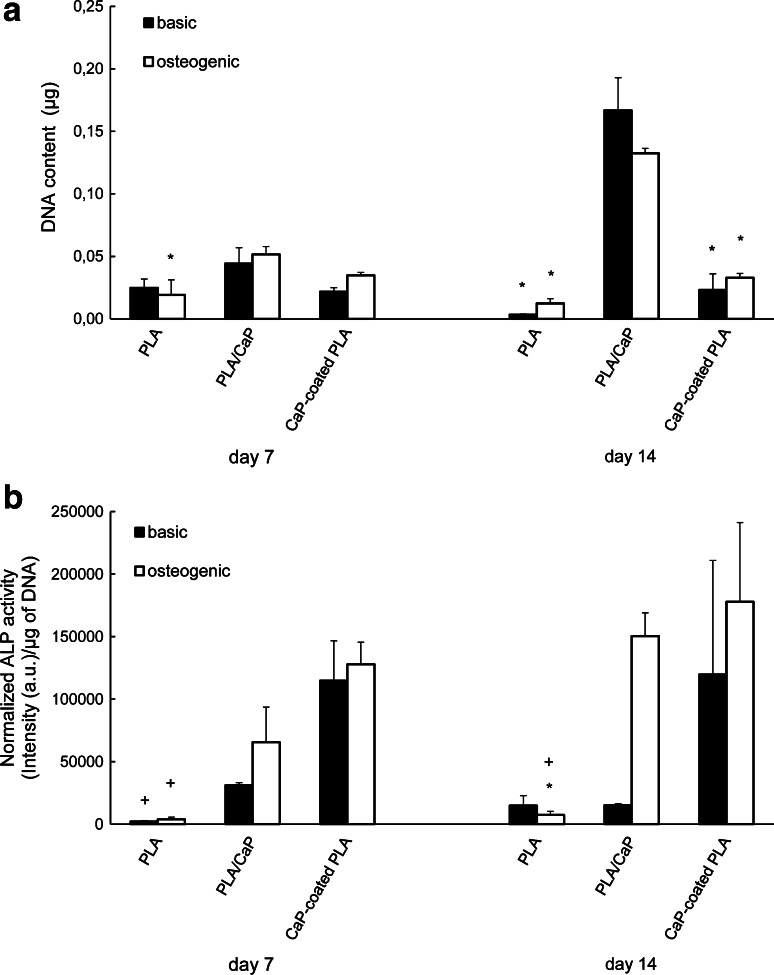
DNA content (**a**) and ALP activity (**b**) of hMSCs cultured on PLA, PLA/CaP and CaP-coated PLA particles. DNA content of hMSCs cultured on PLA/CaP particles was higher than that of hMSCs cultured on CaP-coated PLA and PLA particles. The ALP activity of the hMSCs was enhanced when cultured on the two hybrid materials as compared to the PLA control. *Asterisk symbol* indicates *P* < 0.05 when compared to PLA/CaP composite and *plus symbol* indicates *P* < 0.05, when compared to CaP-coated PLA

The DNA content of the hMSCs cultured on the PLA and the two hybrid samples was quantified at days 7 and 14 (Fig. 5a). At day 7, the DNA content of cells cultured on PLA/CaP composite was slightly higher as compared to PLA and CaP-coated PLA conditions in both media, however, the only significant difference was found between PLA/CaP composite and PLA particles, in osteogenic medium. At day 14, hMSCs cultured on CaP-coated PLA particles showed slightly higher DNA content compared to those cultured on PLA particles in both media, although this difference was not statistically significant. hMSCs cultured on composite, however, showed significantly higher DNA content compared to those cultured on PLA and coated PLA particles, both in basic and osteogenic medium. While only small, non-significant temporal increase in the DNA content of hMSCs cultured on PLA and coated PLA was observed, the DNA content of hMSCs cultured on PLA/CaP composite particles increased 3–4 times between days 7 and 14.

ALP activity of the hMSCs was also quantified and normalized for the DNA content (Fig. 5b). At day 7, in both basic and osteogenic medium, both PLA/CaP composite and CaP-coated PLA samples showed higher ALP activity compared to PLA, however, this effect was significant only between CaP-coated PLA and PLA. ALP activity of hMSCs cultured on CaP-coated PLA was slightly higher than when the cells were cultured on PLA/CaP composite. At day 14, in basic medium, ALP activity measured on CaP-coated PLA was measured to be higher, although not significantly, than what was found on PLA and PLA/CaP composite samples. In osteogenic medium, both hybrid materials showed a significantly higher ALP activity than the pure polymer.

**Fig. 6:**
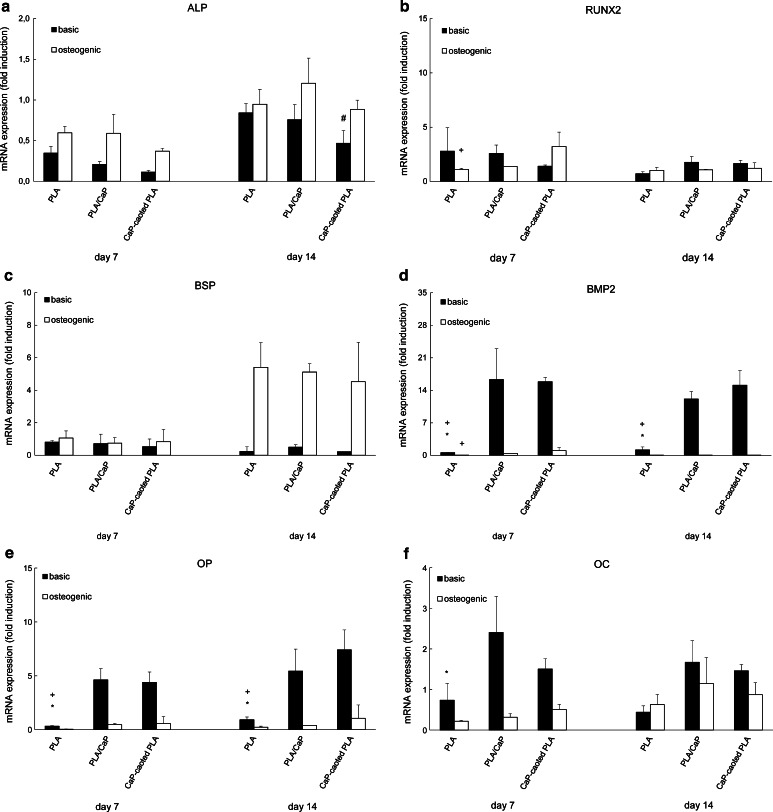
mRNA expression of ALP (**a**), RUNX2 (**b**), BSP (**c**), BMP2 (**d**), OP (**e**) and OC (**f**) in hMSCs cultured on PLA, PLA/CaP and CaP-coated PLA particles. Minor differences were detected in the mRNA expression of ALP, RUNX2 and BSP in hMSCs cultured on different materials. In contrast, the expression of BMP2, OP and OC genes was substantially higher in hMSCs cultured on PLA/CaP and CaP-coated PLA particles in comparison with PLA particles, in particular in basic cell culture medium. *Hash symbol* indicates *P* < 0.05 when compared to PLA, *asterisk symbol* indicates *P* < 0.05 when compared to PLA/CaP composite and *plus symbol* indicates *P* < 0.05, when compared to CaP-coated PLA

The mRNA expression of a panel of osteogenic markers including ALP, RUNX2, BSP, BMP2, OP and OC by hMSCs cultured on the three material types in either basic or osteogenic medium was analyzed after 7 and 14 days (Fig. 6a–f). No significant differences in the ALP mRNA expression were observed among cells cultured on different materials, except at day 14 in basic medium, where PLA particles exhibited a higher ALP mRNA level as compared to the two hybrid materials. In general, the expression of the ALP gene was low at both time points and in both media, not exceeding a 1.5-fold change. At day 7, a significantly higher level of RUNX2 mRNA was measured on CaP-coated PLA as compared to PLA particles without CaP in osteogenic medium, while no differences were found at day 14. At both time points, the expression of BMP2 and OP was higher in the hMSCs cultured on the two CaP-containing materials as compared to the cells cultured on PLA particles in basic medium. mRNA level of these two markers in osteogenic medium was in general low, however, a significant difference was observed in the expression of BMP2 between CaP-coated PLA and PLA particles at day 7. The mRNA level of OC was also higher in hMSCs cultured on CaP-containing particles compared to those cultured on PLA particles at both time points and in both media, although the only significant difference was observed between CaP-coated PLA and PLA particles at day 7 in basic medium.

## Discussion

In a search for successful synthetic alternatives to natural bone grafts, hybrid biomaterials are developed in which desired properties of the individual components are combined. In the current study, we have compared two hybrid materials consisting of CaP and PLA and investigated whether the method of combining the two, affects the response of hMSCs. While CaPs are widely used as bone graft substitutes owing to their resemblance to bone mineral [7] and related biocompatibility and bioactivity [1], polymers offer more diversity when it comes to mechanical properties [15–17].

An amorphous PLA with a relatively low molecular weight was selected for this study. PLA, being an aliphatic polyester, degrades by hydrolysis and the degradation profile of the polymeric phase has been shown to affect the degradation of hybrid materials, such as PLA/CaP composites [18]. Barbieri et al. [20] studied the role of the PLA molecular weight on mechanical and physicochemical properties of PLA/HA composite, similar to the one used in this study, as well as on their bone forming ability in vivo. The results revealed a higher fluid uptake by the composite prepared using low molecular weight PLA, resulting in release of more Ca^2+^ and Pi ions from and faster degradation of the composite. Moreover, heterotopic bone formation, or osteoinduction, which is considered an important characteristic of bone grafts and their substitutes, was only detected in the composites prepared with low molecular weight PLA.

To prepare monolithic composite in the current study, equal amounts of nano-sized CaP powder and PLA were mixed and extruded, a method that allowed preparation of a homogenous composite with a relatively high ceramic content. The TGA results demonstrated a mass loss of 56 % in the composite material within the temperature range of 200–300 °C. Within this temperature range, the organic component of the composite is expected to be completely thermally degraded and removed. No significant mass loss was observed at higher temperatures, indicating a total ceramic content to be 44 %, which correlated well with the initial HA/PLA weight ratio used to produce the composite material. CaP content of PLA/CaP composites has been shown to substantially influence the mechanical properties of the composite [43–45]. On the other hand, the CaP content is also a critical factor determining degradation rate, bioactivity and bone forming ability of the composite, showing that indeed the properties of the individual components determine the properties of the hybrid material. Results of intramuscular implantation of PLA/HA composites with HA content varying from 0 to 40 wt% in dogs showed that heterotopic bone formation only occurred in the composite containing 40 wt% of HA [19]. Heterotopic bone formation was also observed upon implantation of HA/PLA composite with weight ratio of 50/50 prepared by extrusion technique in sheep and dog in vivo models, while no bone formation was observed in PLA control implants [18, 20].

A biomimetic method, involving immersion of polymeric particles into a solution containing Ca^2+^ and Pi ions under near-physiological conditions was used to deposit a homogenous layer on the polymer surface that was a mixture of OCP and apatite. Both these phases are biologically relevant, with carbonated apatite closely resembling the composition of bone mineral [7] and OCP being suggested as a precursor of biological apatite in hard tissue [46]. Both phases are also less stable than hydroxyapatite, showing a faster dissolution at neutral pH values [47]. In contrast to a relatively high CaP content of the monolithic composite, the TGA data showed that in CaP-coated particles, the mineral phase represented only 6.2 wt%.

Interestingly, despite this large difference in total mineral content between the two hybrid materials, upon immersion in SPS, a buffered solution, both materials showed a maximum release of approximately 200 µM and 100 µM of Ca^2+^ and Pi ions, respectively. Nevertheless, this concentration was more rapidly reached in CaP-coated PLA than in PLA/CaP composite, which can be related to both difference in CaP phase and the higher exposure of the CaP phase to the solution. While in the CaP-coated PLA, all CaP is on the surface, in the composite material, the majority of the mineral is in the bulk, and release of ions is dependent on the polymer degradation and the diffusion of the ions through the material bulk. In contrast to what was observed in SPS that, as-prepared, does not contain Ca^2+^ or Pi ions, in cell culture medium, a direct decrease of Ca^2+^ concentration was observed upon immersion of PLA/CaP composite particles or CaP-coated PLA particles, plausibly suggesting precipitation of a new CaP phase as was observed previously [48].

Regarding the differences in response of hMSCs to the two hybrid materials, with PLA without CaP as a control, cell proliferation on PLA/CaP composite particles was higher as compared to PLA. Previous results have shown that supplementing cell culture medium with 4, 7.8 or 8 mM Ca^2+^ increased the DNA content of hMSCs cultured on tissue culture plastic [49, 50], suggesting a positive effect of Ca^2+^ ions on hMSCs proliferation. In one of our previous studies [18], extruded PLA and PLA-HA composite with similar characteristics as used here were casted into pellets with a smooth surface. In contrast to the present study, no differences in proliferation of hMSCs were observed between the polymer and composite material, which may be due to differences in total surface area between a 2D pellet and a 3D environment of a collection of particles. Furthermore, differences between the two studies could, for at least in a part, be caused by the physical differences between materials, such as the observed surface roughness.

Besides differences in proliferation of hMSCs as a result of presence of CaP, differences were also observed dependent on the method used to combine the polymer and the ceramic phase. Cells cultured on PLA/CaP composite particles had a higher DNA content as compared to those cultured on CaP-coated PLA particles. Both differences in the chemical composition and topographical properties between these two materials may be responsible for this effect. This is in accordance with previous studies showing a more pronounced osteoblast attachment to an HA surface as compared to an OCP surface. Besides the difference in chemical composition and degradation properties, these two materials exhibited different grain size, which could also be of influence on cell attachment [51].

The ALP activity of hMSCs cultured on either PLA/CaP composite or CaP-coated PLA was higher as compared to that of cells cultured on PLA particles. Similarly, other studies also demonstrated a higher ALP activity of various cell types when cultured on PLA/CaP composite materials prepared using different techniques, in comparison with the polymer not containing the mineral phase [18, 52–54]. Furthermore, Kim et al. [55] and Kung et al. [56] also observed that the ALP activity of bone marrow- and adipose-derived mesenchymal stem cells cultured on fibrous PLA meshes, the surface of which was covered with CaP, was significantly higher as compared to the culture on PLA samples without the ceramic. Danoux et al. [50] observed that the addition of 4 or 8 µM Ca^2+^, or 4 µM Pi ions to the cell culture medium enhanced the ALP activity of hMSCs. Furthermore, it was also shown that the individual release of these ions from only calcium-containing or only phosphate-containing PLA particles increased the ALP activity of hMSCs compared to pure PLA particles. These results suggested that both Ca^2+^ and Pi ions released from the particles were responsible for promoting ALP activity in hMSCs.

Surprisingly, no effect of the hybrid materials on the mRNA level of ALP was detected, which may be explained by a transient peak expression of mRNA, in contrast to proteins, which are more stable. Similar to ALP, RUNX2 and BSP genes were not influenced at mRNA level when the cells were cultured on different types of particles.

The expression of BMP2, OP and OC genes was substantially increased in hMSCs cultured on the PLA/CaP composite and CaP-coated PLA particles as compared to those cultured on PLA particles not containing CaP. These findings are in line with previously published studies, where the presence of CaP on electrospun PLA meshes had a positive effect on the expression of osteogenic markers such as collagen type I, OC and OP on stem cells from different origin [55, 56]. Furthermore, it was previously shown that the expression of BMP2, OP and OC markers wee significantly enhanced at elevated Ca^2+^ levels [49, 50]. Although the Ca^2+^ level in the cell culture medium was shown to decrease in the presence of CaP-containing particles, it should be noted that the ionic concentration was measured in the bulk medium, not taking into account possible concentration differences between the bulk and the medium in close vicinity of the particle surface where cells are found, where Ca^2+^ concentration may be higher. Furthermore, apart from this chemical effect, the effect of physical surface properties of these materials, like roughness, surface area, etc. on differentiation of hMSCs should not be neglected, although these effects were not separately studied here.

No significant differences were observed in the expression of the osteogenic markers between hMSCs cultured on PLA/CaP composite and those cultured on CaP-coated PLA. The two hybrid materials differed in several properties including the phase, the amount and the availability in the CaP component, as well as the physical surface properties, which, taken together, resulted in a more pronounced decrease in concentration of Ca^2+^ and, expectedly Pi ions in cell culture medium in the case of the monolithic composite as compared to the CaP-coated PLA. These differences however, were not reflected in the differentiation potential of hMSCs when cultured on the different materials suggesting that both methods of combining PLA and CaP can be used to enhance the osteogenic differentiation of hMSCs as compared to PLA without CaP.

## Conclusion

The results of the current study confirmed the beneficial effect of CaP regarding the osteogenic differentiation of hMSCs. This effect was independent of the method used to combine the CaP and the PLA component into a hybrid material, i.e. generation of a monolithic composite or coating of the polymer with a ceramic layer.
